# Direct Percutaneous Abdominal Venous Access for Endovascular Therapy

**DOI:** 10.1016/j.jvsv.2025.102309

**Published:** 2025-09-01

**Authors:** Mohammad A. Amarneh, Kyung Rae Kim, Mohammed H. Alomari, Ahmad I. Alomari

**Affiliations:** Division of Vascular and Interventional Radiology, Boston Children's Hospital and Harvard Medical School, Boston, MA

**Keywords:** Vascular malformations, Mesenteric veins, Pelvic arteriovenous malformations, Direct venous access, Ultrasound guidance, Embolization

## Abstract

**Objective:**

To evaluate the feasibility, safety, and clinical applications of ultrasound-guided direct percutaneous access to ectatic abdominal veins for the embolization of vascular malformations.

**Methods:**

Medical records, imaging studies, and procedural details were retrospectively reviewed for patients who underwent embolization procedures for vascular malformations with ultrasound-guided percutaneous access to intra-abdominal veins, including the pelvic, retroperitoneal, and portomesenteric veins.

**Results:**

A total of 38 direct percutaneous vein accesses were performed across 25 procedures in 9 patients (age range, 3-58 years). Access sites included retroperitoneal veins (n = 12), dilated and tortuous internal iliac vein branches (n = 8), the superior mesenteric vein (n = 8), the inferior mesenteric vein (n = 1), ileocolic vein (n = 8), and right colic vein (n = 1). Catheter sizes ranged from 3F to 5F. All procedures were technically successful. Seven minor access-related complications occurred; all were managed conservatively.

**Conclusions:**

Ultrasound-guided percutaneous access to dilated intra-abdominal veins is feasible and associated with minimal morbidity. It offers a valuable alternative for patients with complex vascular malformations requiring access to deep abdominal veins.

**Article Highlights**

• **Type of Research:** Single-center, retrospective, observational study
• **Key Findings:** Conventional methods for accessing the deep intra-abdominal veins can be challenging for various reasons. Direct percutaneous cannulation of deep intra-abdominal veins, such as the mesenteric, pelvic, or retroperitoneal veins, using ultrasound guidance may offer safe and efficient alternative access for diagnostic and therapeutic purposes. Direct access can be obtained using various needles and catheters with minor morbidity.
• **Take Home Message:** Ultrasound-guided direct percutaneous access to dilated intra-abdominal veins—including the pelvic, retroperitoneal, and portomesenteric veins—is a feasible and safe technique that enables effective embolization of complex vascular malformations when conventional access routes are limited or unsuccessful. This approach provides real-time visualization, stable catheter positioning, and minimal morbidity and should be considered a valuable alternative in challenging cases.

Deeply seated intra-abdominal and pelvic veins are generally not suitable for direct percutaneous access owing to the presence of overlying arteries, bowel, and viscera, which pose significant risks for procedure-related complications. Conventional access to abdominal systemic veins is typically achieved via a retrograde approach, whereas the portomesenteric veins are accessed through transhepatic or trans-splenic routes.

Safe and stable percutaneous access to intra-abdominal veins can be challenging, particularly in cases of markedly dilated and tortuous pelvic veins, postembolization changes, or chronic thrombosis of the portal or splenic veins. When conventional access methods fail, direct percutaneous access may provide a safe and effective alternative.

Arteriovenous malformations (AVMs) are high-flow vascular malformations in which arterial blood is shunted directly into veins through a dysplastic nidus, bypassing the capillary bed. This abnormal circulation increases venous pressure and flow, leading to vascular dilation, tissue ischemia, congestion, and complications such as ulceration, infection, bone destruction, hemorrhage, or even high-output cardiac failure.^1^ In contrast, venous malformations are low-flow vascular malformations involving abnormal venous wall development without arteriovenous shunting. These differences in flow and structure result in distinct clinical courses and require different therapeutic strategies.^2^

The general principle of AVM management is nidus obliteration, most often achieved with ethanol embolization to induce endothelial destruction and thrombosis. In large or high-flow fistulous AVMs, coil embolization is a valuable adjunct, deployed in major outflow veins or fistulas to reduce flow and pressure. This staged approach—coiling followed by ethanol embolization—improves safety and efficacy by limiting nontarget ethanol embolization, lowering systemic toxicity, and allowing effective nidus eradication within safe dose limits.^3^

This study reports a cohort in which ultrasound-guided direct percutaneous access was used as an alternative route to access dilated and tortuous pelvic, retroperitoneal, and mesenteric veins for diagnostic and interventional purposes, demonstrating its feasibility and potential clinical applications.

## Methods

The study was approved by the Committee on Clinical Investigation at Boston Children's Hospital (#M09030158), and the requirement to obtain an informed consent was waived. This study was conducted in accordance with the Declaration of Helsinki.

Medical records, imaging studies, and procedural details were retrospectively reviewed for patients who underwent percutaneous direct access of dilated and tortuous abdominal and pelvic veins. Procedure-related complications were documented and categorized according to the Society of Interventional Radiology classification system for complications by outcome.^4^ The coagulation profile, including complete blood count, prothrombin time, international normalized ratio, partial thromboplastin time, fibrinogen and D-dimer, was obtained before the procedure. Any significant coagulopathy was corrected, and anticoagulation medications were appropriately discontinued before the procedure. All procedures were performed under general anesthesia and standard sterile conditions.

Preprocedural cross-sectional imaging, such as contrast-enhanced computed tomography (CT) or magnetic resonance imaging, was used—along with angiography and intraprocedural ultrasound examination—to assess venous anatomy and complexity; evaluate the feasibility of arterial, retrograde, or direct access approaches; and identify intervening structures to plan the optimal path for direct access when required.

**Fig 1:**
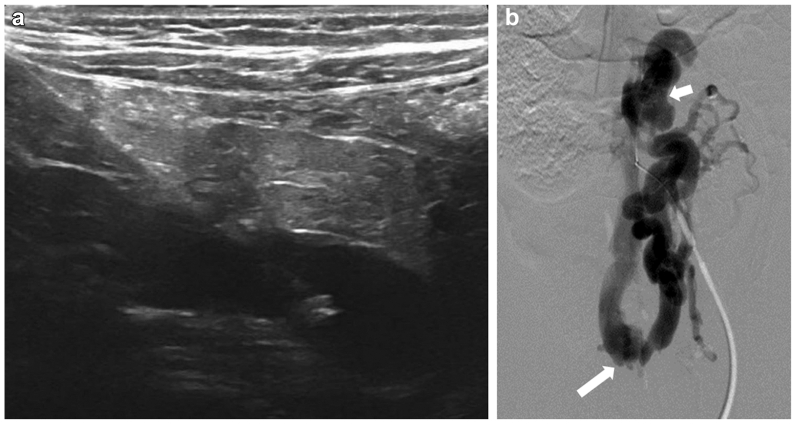
Patient 2 had a history of portosystemic shunt requiring portal pressure measurement before surgical ligation. **(A)** Ultrasound image depicting the needle placed via the abdominal wall into dilated inferior mesenteric vein. **(B)** Portomesenteric venography showing dilated inferior mesenteric vein (*short arrow*) with retrograde flow to the right internal iliac vein (*long arrow*).

**Fig 2:**
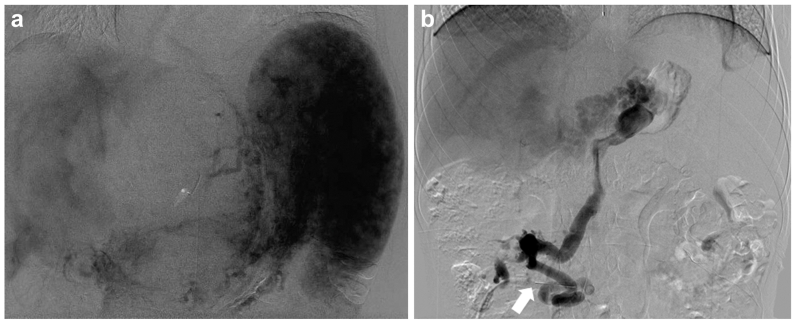
Patient 1 had a history of portomesteric thrombosis requiring portal pressure measurement before splenorenal shunt surgery. **(A)** Venous phase celiac arteriography showed faint opacification of the gastric and intestinal draining veins with no visualization of the main portomesenteric veins. Note the displacement of the stomach and liver by the large upper abdominal venous malformation and splenomegaly. **(B)** Venography through direct access depicting dilated superior mesenteric vein terminating in portal vein collaterals without opacification of the main portal vein.

**Fig 3:**
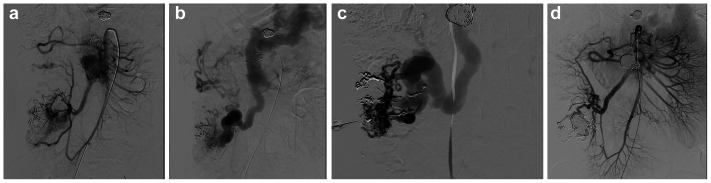
Patient 4 had a complex ileocolic arteriovenous malformation (AVM). Angiography in the arterial **(A)** and venous **(B)** phases demonstrating a complex arteriovenous fistula between dilated ileocolic and right colic arteries and aneurysmal ileocolic vein. **(C)** Venography via direct access demonstrating the complexity of the ileocolic draining vein. **(D)** Postembolization angiography showing no residual arteriovenous shunting.

Target veins were accessed under ultrasound guidance through the anterior abdominal wall, avoiding bowel loops and arteries. For diagnostic evaluation of the mesenteric veins, small needles: 21G EchoTip needle (Cook Medical) or 24G MicroSlide needle (Galt Medical) were advanced and exchanged over a 0.014 to 0.018″ guidewire for a 3F vascular introducer. Venographic and hemodynamic assessment of the portomesenteric venous system was then performed (Figs 1 and 2). In the case involving embolization of a mesenteric AVM, superior mesenteric vein angiography was performed via standard femoral artery access both before and after embolization. Access to the immediate mesenteric draining veins was achieved using 3F to 5F catheters, with or without a coaxial microcatheter, to perform embolization (Fig 3).

**Fig 4:**
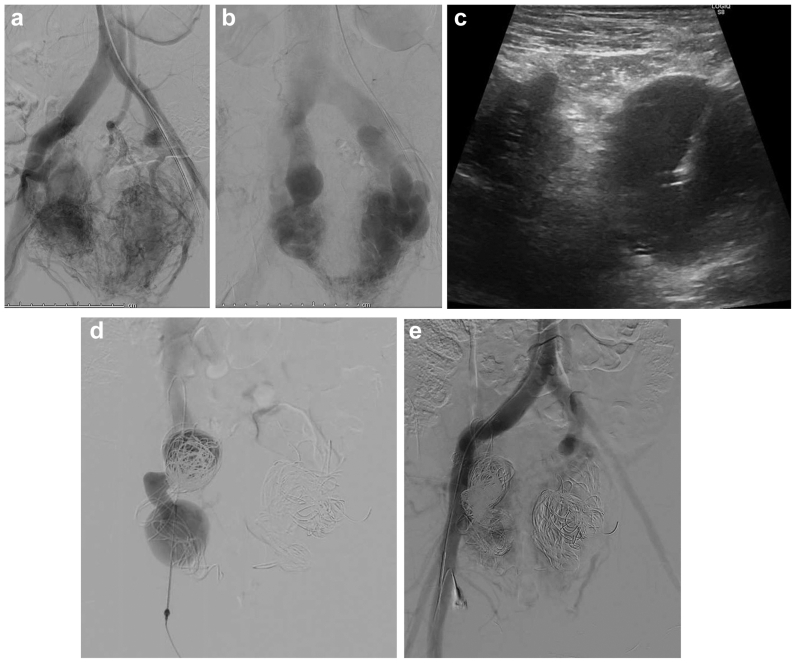
Patient 5 had a complex pelvic arteriovenous malformation (AVM). **(A** and **B)**. Arterial and venous phases of pelvic angiography demonstrating large bilateral pelvic arteriovenous shunts with numerous arterial feeders into markedly ectatic draining tributaries of the internal iliac veins. **(C)** Ultrasound guidance for direct access of an immediate draining vein using a 19G trocar needle. **(D)** Venography demonstrating large iliac aneurysmal immediate draining vein. Note the large embolic nests from prior procedures. **(E)** Postembolization angiogram revealing a marked reduction of the arteriovenous shunting.

In cases of markedly dilated tributaries of the internal iliac veins associated with large pelvic AVMs, standard femoral arterial access and angiography were initially performed. Retrograde transfemoral cannulation was used for angiography and embolization of the immediate draining veins, which were tributaries of the internal iliac veins (Fig 4). Retrograde transvenous access was sometimes challenging owing to tortuous anatomy, unfavorable angles from femoral or jugular approaches, and the complex angioarchitecture of markedly dilated draining veins (Fig 4). In such cases, selective cannulation of the target vein was time consuming or not achievable, which increased procedural and radiation times. Direct percutaneous access was therefore used to achieve stable catheterization and effective embolization.

For direct percutaneous access, 21G EchoTip, 20G Chiba, or 18G trocar needles (Cook Medical) were used. The needle was exchanged over a 0.018″ to 0.035″ wire for a 3F inner dilator of MAK-NV inner dilator (Merit), or 4F stiffened Micropuncture sheath (Cook Medical), then further exchanged for a 4F to 5F diagnostic catheter over a 0.035″ hydrophilic Glidewire wire (Terumo Medical). Under fluoroscopic guidance, the catheter was navigated into the target vein. Venography and embolization of the large draining veins were performed through either the catheter or trocar needle. Postembolization angiography was conducted to confirm treatment efficacy.

The access points were closed using manual compression for approximately 5 minutes. No closure device or tract embolization was used.

## Results

A total of 38 percutaneous direct venous accesses were successfully obtained during 25 procedures performed in 9 patients (age range, 3-58 years; 4 females, 5 males). Each procedure involved one to four accesses of dilated, tortuous intra-abdominal and pelvic veins. The clinical and technical details are summarized in the Table. The clinical diagnosis included large slow-flow vascular malformation (n = 3), large pelvic AVMs (n = 3), large retroperitoneal AVMs (n = 2), and complex mesenteric AVMs (n = 1). The accessed veins included the large tributaries of the internal iliac vein (n = 8), retroperitoneal draining vein (n = 12), superior mesenteric vein and its branches (n = 8), inferior mesenteric vein (n = 1), ileocolic vein (n = 8), and right colic vein (n = 1). The maximal diameter of the accessed veins ranged from 7 to 65 mm.

**Table:** Summary of demographics, clinical features, and procedural and technical details

Patient	Gender	Age, years, weight, kg	Diagnosis	Procedure	Indication for the direct access	Vein, maximal diameter	No. of accesses, needle used	Catheters	Embolic tools
1	Male	18, 70	Massive mediastinal, abdominal and retroperitoneal venous malformation. Portomesenteric thrombosis, GI bleeding	I	Poor opacification of portomesenteric veins during arteriography before surgical splenorenal shunt; need to measure portal pressure	SMV, 33 mm	1, 21G	3F vascular introducer	None
2	Male	3, 18	Lymphatic and venous malformations in the right lower extremity and pelvis; GI bleeding, Portal hypertension	I	Portal study before ligation of dilated IMV and portosystemic shunt; need to measure portal pressure	IMV, 12 mm	1, 24G	3F vascular introducer	None
3	Male	13, 73	Lymphatic and venous malformations in the left lower extremity, pelvis and retroperitoneum; GI bleeding	I	Chronic portomesenteric thrombosis; need to measure portal pressure	SMV tributary, 8.5 mm	1, 21G	3F vascular introducer	None
4	Female	25, 81	PIK3CA-related overgrowth syndrome with complex mesenteric AVM, portal vein thrombosis, portal hypertension, thrombocytopenia, and splenomegaly	I	Embolization of a large complex mesenteric AVM with several aneurysmal immediate draining veins; no straightforward access or transhepatic portal access to the immediate draining veins	Ileocolic vein, 8 mm	1, 21G	5F Kumpe	0.020 Penumbra and Ruby detachable coils
				II		#1: Right colic vein (3 mm) #2: SMV aneurysmal, 45 mm	#1: 21G #2: 19G	#1: 3F Bernstein #2: 2.95F Px Slim microcatheter coaxially introduced through the 19G needle	0.020 Penumbra detachable coils, n-BCA glue
				III		Ileocolic vein, 8 mm	1, 20G needle	4F C2 catheter, coaxial Renegade STC microcatheter	0.020 Penumbra detachable coils, n-BCA glue
				IV		#1: Ileocolic vein #2: SMV aneurysmal branch 45 mm	2, 21G	4F Vert catheter, coaxial Renegade STC	0.018 Interlock IDC coils, 0.020 Penumbra detachable coils
				V		Ileocolic vein branches (×4), 7, 8, 11, and 18 mm	4, 20- and 21G needles	3F Bernstein, 4F Kumpe, 4F Vert	0.018 Interlock IDC coils, 0.020 Penumbra detachable coils, n-BCA glue
				VI		#1 1: SMV aneurysmal branch: 45 mm #2: Ileocolic vein branch, 8 mm.	2, 21G needle	4F Kumpe, coaxial Renegade STC	0.018 Interlock IDC coils
				VII		SMV aneurysmal branch, 40 mm	1, 21G needle	3F Bernstein	0.020 Penumbra detachable coils
5	Male	58, 133	Large left pelvic AVM	I	Embolization of left pelvic draining veins after transvenous embolization	Left IIV, 33 mm	1, 21G	4F Kumpe 5F MPA 5F vertebral, Coaxial 5F Envoy guiding +4F vertebral	MReye and Nester coils Core movable 0.035″ Bentson wires, n-BCA glue
6	Female	31, 42	Massive bilateral pelvic and right lower extremity AVM; heart failure	I	Embolization of left pelvic draining veins following transvenous embolization	Left IIV, 33 mm	1, 21G	4F Kumpe	Core movable Bentson 0.035″ wire
				II	Embolization of right pelvic draining veins during sclerotherapy procedure	Right IIV, 42 mm	1, 20G	5F Kumpe	MReye and Nester coils; Core movable Bentson 0.035″ wire, glue
7	Female	23, 60	Large retroperitoneal AVM	I	Embolization of extensive right retroperitoneal draining vein	Large retroperitoneal draining veins 8-9 mm	4, 18G	Access 1-3: none Access 4: coaxial Rapid Transit microcatheter	Ruby 0.020″ coils, Penumbra 0.020”coils, n-BCA glue
				II	Embolization of extensive right retroperitoneal draining vein	Large retroperitoneal draining vein 8-9 mm	2, 20G	Access 1: MAK-NV 3F inner dilator Access 2: 4F vascular introducer	n-BCA glue
				III	Embolization of extensive right retroperitoneal draining vein	Large retroperitoneal draining vein 8-9 mm	4, 18G	Access 1: MAK NV 3F inner dilator Access 2-3: needle only 4: Coaxial Rapid Transit microcatheter	Ruby 0.020″ coils, n-BCA glue
				IV	Embolization of extensive right retroperitoneal draining vein	Large retroperitoneal draining vein 8-9 mm	1, 20G	MAK NV 3F inner dilator	0.020″ Ruby coils, Penumbra 0.020″ coils
				V	Embolization of large right retroperitoneal draining vein	Large complex retroperitoneal draining vein 8-9 mm	1, 20G	MAK NV 3F inner dilator, 4F Vertebral	Penumbra 0.020″ coils, Nester 0.035″ coils
8	Female	30, 43-48	Large complex pelvic AVM	I	Embolization of large right pelvic AVM.	Right internal iliac aneurysmal vein, 14-16 mm	1, 20G	5F Kumpe	Core movable Bentson 0.035″ wire, 0.035″ Nester coils, 0.035″ MReye coil, n-BCA glue
				II	Embolization of a large right pelvic AVM	Right internal iliac aneurysmal vein, 13 mm	1, 21G	4F micropuncture sheath, 4F Kumpe	Core movable Bentson 0.035″ wire, n-BCA glue
				III	Embolization of a large left pelvic AVM	Left internal iliac aneurysmal vein, 15 mm	1, 18G	None	Core movable Bentson 0.035″ wire, 0.035″ Nester coils
				IV	Embolization of large right pelvic AVM	Right internal iliac aneurysmal vein, 13 mm	1, 18G	4F Kumpe	n-BCA glue, Core movable Bentson 0.035″ wire
				V	Embolization of large right pelvic AVM	Right internal iliac aneurysmal vein, 16 mm	1, 18G	4F Kumpe	Core movable Bentson 0.035″ wire
9	Male	23, 123	Large complex retroperitoneal AVM	I	Embolization of large right retroperitoneal venous sac	Large right retroperitoneal venous sac, 40 mm	1, 18G	Direct embolization through the needle, followed by coaxial placement of 2.8F Renegade Hi Flo microcatheter used for additional embolization	0.035″ Nester coils, 0.020 Ruby coils, Core movable Bentson 0.035″ wire, n-BCA glue
				II	Embolization of large right retroperitoneal venous sac	Large right retroperitoneal venous sac, 45 mm	1, 22G	Needle exchanged for an 18G trocar used for embolization	0.035″ Nester coils, 0.020 Ruby coils, Core movable Bentson 0.035″ wire

*AVM,* Arteriovenous malformation; *GDC,* guglielmi detachable coils; *IIV,* internal iliac vein; *IMV,* inferior mesenteric vein; *INR,* international normalized ratio; *n-BCA,* n-butyl-2-cyanoacrylate; *PSPG,* portosystemic pressure gradient (mmHg); *PTT,* partial thromboplastin time; *SMV,* superior mesenteric vein.

Mild coagulopathy, not requiring correction, was noted in four patients, including thrombocytopenia, hypofibrinogenemia, elevated international normalized ratio, partial thromboplastin time, and D-dimer. In two patients, anticoagulation (enoxaparin in patient 2 and oral rivaroxaban in patient 5) was discontinued before the procedure.

The procedures involving access of the mesenteric veins were achieved with one attempt each. Portal pressures were successfully measured in all these patients. The eight accesses into the internal iliac venous tributaries were used for angiography and successful embolization of markedly dilated and tortuous draining veins in pelvic AVMs. Additional accesses, repositioning of needles, and unsuccessful attempts were documented in three procedures. Various embolic agents were safely deployed within the target veins including 0.018” interlock coils (Boston Scientific), 0.020” Penumbra coils (Penumbra), 0.035″ stainless steel core movable wire (Cook Medical), large 0.035″ Nester, and 0.038″ MReye pushable fibered coils (Cook Medical) and n-butyl-2-cyanoacrylate glue (Histoacryl; B. Braun).

**Fig 5:**
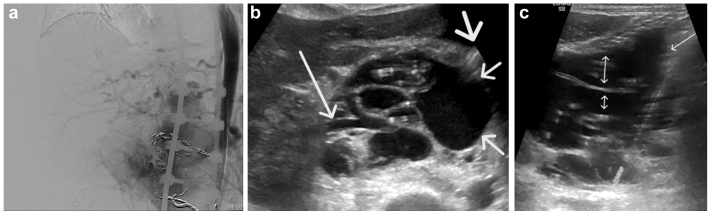
Patient 7 had a complex retroperitoneal arteriovenous malformation (AVM). **(A)** Abdominal angiogram demonstrating extensive retroperitoneal arteriovenous shunting centered around the right renal hilum. **(B)** Transverse ultrasound image demonstrating the complex right retroperitoneal arteriovenous shunt posterolateral to the inferior vena cava (IVC) (*thin arrows*). Note the needle approaching from the left (*thick arrow*), and common hepatic artery traversing the malformation (*long arrow*). **(C)** Transverse ultrasound image demonstrating the transcaval course of the needle. Note tenting of the anterior wall of the IVC and large veins directly draining into the IVC (*double arrow heads*).

Transcaval access of large retroperitoneal draining veins of an extensive retroperitoneal AVM was obtained in five separate procedures in one patient (total of 12 accesses) (Fig 5). The access was obtained through the anterior abdominal wall under ultrasound guidance (patient 7 on the table). Multiple attempts to obtain secure access to the enlarged retroperitoneal draining veins through the right femoral vein access and through the right internal jugular vein access using several combinations of wires and catheters were not successful; thus, the decision was made to obtain direct transabdominal transcaval access. Bowel injury was avoided through meticulous preprocedural path planning using cross-sectional imaging to identify a safe needle trajectory. During the procedure, continuous real-time ultrasound guidance was used to visualize the needle at all times and ensure avoidance of interposed bowel loops.

Seven minor complications related to the direct venous access portion of the procedures were observed. One patient undergoing direct percutaneous access for pelvic AVM embolization developed two small contained pelvic hematomas: one required aspiration of 20 mL of blood during the procedure. Another patient with a complex mesenteric AVM developed a small hemoperitoneum after access to ileocolic and superior mesenteric vein branches in four of seven procedures. Blood was aspirated at the end of each procedure, with volumes ranging from 4 to 35 mL (4, 5, 15, and 35 mL). No further hemorrhage was detected by ultrasound examination after access removal or during the recovery period. The seventh complication involved inadvertent puncture of the right renal artery by a needle and 3F inner dilator of a MAK-NV set in a patient with a large retroperitoneal AVM. The transcaval access was removed without significant bleeding or hematoma formation. A repeat right renal artery angiogram demonstrated no visible arterial injury.

## Discussion

The ability to establish safe and stable access is paramount to the success of any endovascular procedure, particularly in anatomically challenging or dilated and tortuous vessels. Direct percutaneous cannulation may offer safe and efficient access to the blood vessels. Nevertheless, such an approach to deeply located veins (eg, pelvic or portomesenteric veins) is usually avoided owing to the risk of bleeding and injury of adjacent organs. This approach should be considered when standard retrograde or arterial routes are limited by complex, tortuous venous or arteriovenous anatomy and when a direct percutaneous path can be planned and executed safely.

Transvenous embolization of large pelvic AVMs with dominant venous drainage can be achieved by the retrograde transvenous approach with good therapeutic outcomes.^5^, ^6^, ^7^, ^8^, ^9^ The direct percutaneous route for accessing deeply located pelvic AVMs can be difficult, with a potential risk for bleeding from large, arterialized veins.^5^^,^^6^ If the retrograde transvenous route fails, directly puncturing the veins is a feasible option. Cho et al^5^ used direct access using 18G or 21G long needles in 4 of 12 patients with pelvic AVM, and Conway et al^7^ only used direct access in 1 of 4 patients. Direct access to pelvic AVM can be achieved under fluoroscopic or CT guidance^5^^,^^8^; however, this approach is not in real time and may make it challenging to accurately target the desired spot among other dilated vessels while avoiding moving bowel loops. In this cohort, all pelvic, retroperitoneal, and mesenteric venous accesses were performed under ultrasound guidance. We believe that ultrasound guidance offers key advantages, including real-time needle visualization, identification of adjacent vessels and viscera, and enhanced ability to safely access veins that are abnormally positioned or otherwise challenging to reach using conventional techniques.

Direct percutaneous access may provide a shorter approach with better stability. For instance, when deploying long or relatively stiff embolic agents (such as core-removed wires or MReye coils), the use of the blunt trocar needle may provide the needed stability and prevents the kickback of softer diagnostic catheters. Access can also be exchanged for different types or coaxial combinations of diagnostic catheters, which can be maneuvered into the nearby vessels in an antegrade or retrograde fashion. Concurrent accesses may also be used for added safety in the deployment of embolic agents.

Percutaneous access to the portomesenteric veins remains a significant technical challenge. In current interventional practice, direct percutaneous puncture of the mesenteric venous system is often limited by anatomical constraints, such as intervening bowel loops and the deep location of these veins within the mesentery.^10^ As a result, access to these veins is more commonly achieved through transhepatic, trans-splenic, or transjugular intrahepatic routes.

Although limited, several case reports and series describe safe percutaneous access of mesenteric branches as alternatives to traditional routes. Onishi et al^11^ reported direct percutaneous puncture of the middle colic vein to embolize a variceal bleed in a patient with portal vein tumor thrombus. Similarly, Ono et al^12^ and Kariya et al^13^ documented direct percutaneous access of the superior rectal vein through the greater sciatic foramen using CT fluoroscopy guidance for rectal variceal sclerotherapy. Additionally, Entezari et al^14^ and Farsad et al^15^ reported direct percutaneous access of the mesenteric veins to facilitate portal vein recanalization. Less commonly used techniques include accessing enlarged paraumbilical veins, direct cannulation through surgical exposure, using a previously created shunt, or transjugular retrograde access via a patent ductus venosus.^16^^,^^17^

In our cohort, several risk factors for bleeding during abdominal venous access were present, including hypertensive arterialized pelvic veins, portal hypertension, coagulopathy, and multiple access attempts. Despite these challenges, only five cases of minor pelvic or intraperitoneal bleeding were observed. These were all self-limiting and successfully managed with aspiration at the end of the procedure, without the need for further intervention or postprocedural treatment. These findings underscore the importance of using ultrasound to assess for peritoneal fluid both before and after venous access. Preprocedural scanning establishes a baseline and helps to identify preexisting ascites. If new fluid is detected post procedure, it can indicate intraperitoneal bleeding. Additionally, the presence of ascites may displace intra-abdominal structures away from the abdominal wall, potentially impairing hemostasis at the access site and increasing the risk of bleeding. Therefore, in patients with significant ascites, preprocedural paracentesis should be considered to reduce this risk.

This retrospective study is limited by the small cohort of patients included with specific clinical rationale for using the direct access approach. The accessed veins were dilated and tortuous, which facilitated identification with ultrasound examination and access.

## Conclusions

Direct percutaneous access of dilated intra-abdominal veins was safe and feasible in patients with complex vascular malformations without major morbidity. In selected patients, this technique can be further evaluated as a potential alternative approach for access and intervention on deeply seated systemic and portomesenteric veins.

## Declaration of generative AI and AI-assisted technologies in the writing process

During the preparation of this work, the authors used ChatGPT to grammar-check and improve readability. After using this tool, the authors reviewed and edited the content as needed and take full responsibility for the content of the publication.

## Author contributions

Conception and design: MAA, KK, AA

Analysis and interpretation: MAA, KK, MHA, AA

Data collection: MAA, KK, MHA

Writing the article: MAA, KK, MHA, AA

Critical revision of the article: MAA, KK, AA

Final approval of the article: MAA, KK, MHA, AA

Statistical analysis: Not applicable

Obtained funding: Not applicable

Overall responsibility: MAA

MAA and KK contributed equally to this article and share co-first authorship.

## Funding

None.

## Disclosures

None.
